# Integrin αVβ3 Signaling in the Progression of Osteoarthritis Induced by Excessive Mechanical Stress

**DOI:** 10.1007/s10753-022-01770-6

**Published:** 2022-12-08

**Authors:** Fanglong Song, Xiaoyu Mao, Jun Dai, Bingchen Shan, Zhentao Zhou, Yifan Kang

**Affiliations:** 1 Department of Orthopedics, the Second Affiliated Hospital of Soochow University, Suzhou, Jiangsu 215000 China; 2 Department of Orthopedics, Third Affiliated Hospital of Naval Medical University, Shanghai, 200438 China

**Keywords:** chondrocyte inflammation, DMM, integrin αVβ3, mechanical stress, osteoarthritis, weight-bearing area.

## Abstract

Osteoarthritis (OA) is believed to be linked with cartilage degeneration, subchondral bone sclerosis, and synovial inflammation that lead to joint failure, and yet treatment that can effectively reverse the pathological process of the disease still not exists. Recent evidence suggests excessive mechanical stress (eMS) as an essential role in the pathogenesis of OA. Increased levels of integrin αVβ3 have been detected in osteoarthritic cartilage and were previously implicated in OA pathogenesis. However, the role of integrin αVβ3 in the process of eMS-induced OA remains unclear. Here, histologic and proteomic analyses of osteoarthritic cartilage in a rat destabilization of the medial meniscus model demonstrated elevated expression of integrin αVβ3 as well as more serious cartilage degeneration in the medial weight-bearing area. Furthermore, results of *in vitro* study demonstrated that eMS led to a significant increase of integrin αVβ3 expression and phosphorylation of downstream signaling molecules such as FAK and ERK, as well as upregulated expressions of inflammatory and degradative mediators. In addition, we found that inhibition of integrin αVβ3 could alleviate chondrocyte inflammation triggered by eMS both *in vivo* and *in vitro*. Our findings suggest a central role for upregulation of integrin αVβ3 signaling in OA pathogenesis and demonstrate that activation of integrin αVβ3 signaling in cartilage contributes to inflammation and joint destruction in eMS-induced OA. Taken together, our data presented here provide a possibility for targeting integrin αVβ3 signaling pathway as a disease-modifying therapy.

## INTRODUCTION

Currently, the treatment of osteoarthritis (OA) is mainly to relieve symptoms with pharmacological therapy at early stage, and joint replacement is often required in the late stage after suffering from pain and limited mobility for decades [1–3]. Due to the lack of effective disease-modifying therapy, enormous studies have focused on OA pathogenesis in recent years. It has been generally accepted various factors to be associated with OA pathogenesis, including aging, obesity, joint instability, trauma, mechanical stress (MS), and joint inflammation, among which MS has received increasing attention [4–9].

Recent evidence from both *in vitro* and *in vivo* studies suggests that chondrocytes embedded in the cartilage are sensitive and respond to a variety of MS such as fluid shear stress, cyclic stretch, continuous compressive force, and MS generated by liquid perfusion or compressed air [6, 10–13]. There is increasing evidence that MS affects chondrocyte proliferation, inflammation, metabolism, apoptosis, and even immune response [6, 14]. Physiological MS facilitates extracellular matrix (ECM) synthesis and anti-inflammatory effects of chondrocytes, while excessive MS (eMS) is thought to play an essential role in the pathogenesis of OA [5, 11, 15]. As a classic signaling pathway that mediates inflammation, the nuclear factor kappa-light-chain-enhancer of activated B cells (NF-κB) signaling has been reported to be regulated by mechanical loading [15]. However, the precise molecular mechanisms underlying chondrocyte inflammation by eMS have not been fully elucidated.

Integrins are heterodimeric transmembrane proteins composed of α and β subunits that mediate cell adhesion, ECM organization, mechanosensing, signaling, survival, proliferation, and differentiation [16–18]. These heterodimers can be classified into several groups based on their ligand-binding specificities and signaling properties [19]. Evidence from numerous studies suggests a potential role for integrins in the pathogenesis of OA [17, 18]. Elevated levels of α1β1, α3β1, α2β1, α4β1, αVβ3, and possibly α6β1 have been detected in osteoarthritic cartilage [20]. Among them, integrin *α*V*β*3 plays an extremely important role in signal transduction, differentiation, and proliferation in chondrocytes [18, 20, 21]. Moreover, integrin αVβ3 has been reported to promote multiple processes in OA, such as inflammation, apoptosis, angiogenesis, and bone sclerosis, and its level varies with the degree of cartilage degeneration in OA [17, 22, 23].

Integrins have been reported to percept extraneous stimuli both physically and chemically and transduce them into intracellular signaling [16]. Wang et al. found that inflammatory factors and cartilage metabolites in the OA joint cavity can activate and upregulate the expression of integrin αvβ3, and inhibition or knockout of integrin αVβ3 can reduce the degree of OA [17]. This indicates that the upregulation of integrin αVβ3 could be regarded as the reason as well as the result of OA development. Since a general hallmark of integrin is its mechanosensitivity [24], we propose that eMS activates and upregulates the expression of integrin αVβ3, which leads to the onset and progression of OA.

Destabilization of the medial meniscus (DMM) has been widely used to establish animal OA models [25–29]. The meniscus is often regarded to possess features of shock absorption and load distribution. It has been believed that medial meniscectomy induces changes in joint stability and local stress concentration between the cartilage surface of the femoral condyle and the tibial plateau, leading to cartilage degeneration and wear [30, 31]. Recent studies have shown that MS-induced cartilage degeneration in OA goes far beyond wear and tear. MS could activate several intracellular signaling pathways such as integrin/focal adhesion kinase (FAK), mitogen-activated protein kinases (MAPKs), and NF-κB, thus regulating proliferation, metabolism, and inflammation in chondrocytes [12, 32, 33].

Numerous studies have shown that cartilage damage in the medial part of the knee joint is more severe in a DMM model [17, 26, 27], yet the different activation of signaling pathways between medial and lateral knee chondrocytes has not been fully investigated. The extent of articular inflammation and damage is believed to associate with the severity of OA and local MS conditions [5, 15, 34]. Zhen et al. reported that distribution of TGFβ activity in osteoarthritic articular cartilage is altered because of aberrant mechanical loading in different areas of the joint which is increased in high-stress areas and decreased in low-stress areas [9]. Therefore, the phenomenon that different levels of cartilage degeneration in the two parts result from the different MS that chondrocytes suffered after DMM [30]. It is reasonable to speculate that MS may activate different signaling pathways or a certain signaling pathway in different degrees in the medial and lateral parts of the knee joint.

The hypothesis of the present study was that eMS led to the upregulation of integrin αVβ3 which resulted in the onset and progress of OA. To test this hypothesis, a rat OA model with DMM was used to investigate the effect of different MS conditions on cartilage degeneration by examining integrin αVβ3 as well as OA-related inflammatory and degradative mediators such as collagen II, aggrecan, MMP-9, 13, runt-related transcription factor 2 (Runx2), and a disintegrin and metalloproteinase with thrombospondin type-5 motif (Adamts-5) in the chondrocytes of medial and lateral weight-bearing areas of the knee, respectively. Furthermore, the effect of eMS on chondrocytes *in vitro* was also examined by detecting the mediators mentioned above and the activation of integrin αVβ3-dependent signaling molecules. Moreover, we investigated whether inhibition of integrin αVβ3 could alleviate inflammation triggered by eMS *in vivo* and *in vitro* and verify αVβ3 potentially a cartilage degeneration preventer.

## MATERIALS AND METHODS

### Chemicals and Reagents

Cyclo (RGDyK) (CYC, catalog S7844) was purchased from Selleck chem, USA. Anti-p-ERK (catalog 4377), ERK1/2 (catalog 4695), p-FAK (catalog 3283), FAK (catalog 3285), β-actin (catalog 4970), and HRP-conjugated secondary (catalog 7074) antibodies were purchased from Cell Signaling Tech, USA. DMSO (catalog C6164) and collagenase II (catalog C2-BIOC) were purchased from Sigma, USA.

### Animal Study

All the animal experiments were approved by the Animal Research Ethics Committee of Soochow University (201708A105).

A total of 80 male SD rats weighing 300–350 g were purchased from the Soochow University Animal Administration Center.

Firstly, 16 rats underwent DMM surgery on the right knee joints, and all specimens were harvested at 12 weeks postoperatively and evaluated using histological observation (*n* = 8) and qRT-PCR assay (*n* = 8) to determine the degree of cartilage degradation and differences of gene expressions in the medial and lateral region of the degenerated knees. Then, the last 64 animals that underwent DMM surgery on the right knee joints were randomly divided into two groups: the control group (*n* = 32) and the CYC-treated group (*n* = 32). Animals of the CYC-treated group received an intra-articular injection of CYC (4 mg/kg) per week into the right knee, while 0.1 ml PBS was injected into the right knees of the control group 3 days after DMM for 6 or 12 weeks. Sixteen specimens from each group were harvested at 6 and 12 weeks postoperatively and evaluated using histological observation (*n* = 8) and qRT-PCR assay (*n* = 8), respectively.

For the DMM surgery, all animals were operated under general anesthesia with 3% intraperitoneal sodium phenobarbital (1.0 ml/kg). A middle anterior incision was made on their right knees and followed by medial meniscectomy via an incision on the medial aspect of the joint capsule. Following surgery, all the rats were allowed free activity without immobilization.

### Histological Analysis

Rats were sacrificed by cervical dislocation at 6- or 12-week post-surgery. Knee joints were removed and fixed in 10% formalin solution at 4 °C for 24 h and then decalcified with EDTA for 4 weeks. The decalcified specimens were subsequently dehydrated in alcohol and embedded in paraffin blocks and serial 4-mm thick coronal sections were made. The serial sections including the severely degenerated area were stained with toluidine blue (TB). The stained sections were analyzed to determine the degree of cartilage degradation at the femoral condyle and tibial plateau which was comparable in locations in each group. The histological evaluations were performed independently using light microscopy by two observers in a blinded fashion according to the modified Mankin score system. There were 8 specimens in each group at each time point for histological analysis. Representative histological images matching the conclusions were presented.

### Collection, Isolation, and Culture of Rat Articular Chondrocytes

The cartilage of 1-week-old Sprague–Dawley rats was harvested and minced from knee joints before being digested with 0.25% trypsin (Gibco, USA). The trypsin was then removed, and the cartilage was washed with phosphate-buffered saline (PBS) three times, after which 0.2% collagenase II was added for digestion at 37 ℃ for 4 h. A 200-lm mesh strainer was used to filter the above solution, and the cells were collected by centrifugation and then cultured in Dulbecco’s modified Eagle’s medium (DMEM)-F12 medium (Hyclone, USA) supplemented with 10% fetal bovine serum (FBS, Gibco, USA) and 1% penicillin/streptomycin (Gibco, USA) and incubated with 5% CO_2_ at 37 ℃. The culture medium was changed every other day. The cells were digested and reseed when reached 70–80% confluence as passage 1 (P1). The P1 generation of chondrocytes was used for the experiments in this report. Following starvation in serum-free medium for 12 h, cells were exposed to eMS for 5, 15, or 30 min to explore the effect of MS on the phosphorylation of ERK and FAK in chondrocytes. 10 μM CYC was added to the culture medium [35, 36] or DMSO alone (as vehicle control) for a further 24 h and subsequently exposed to MS for 30 min (cell lysates were measured by Western blot for the kinase assays) or for another 24 h in normal culture medium to investigate expression levels of MMP-9, MMP-13, ADAMTS-5, Runx2, collagen II, and aggrecan with qRT-PCR analyses.

### Cyclic MS Application

When the cells reached 90–95% confluence (about 5 × 10^5^ cells/chamber), the medium was replaced with DMEM/F12 supplemented with 0.5% FBS and antibiotics for 12 h. Following synchronization, cells were exposed to cyclic MS.

**Fig. 1:**
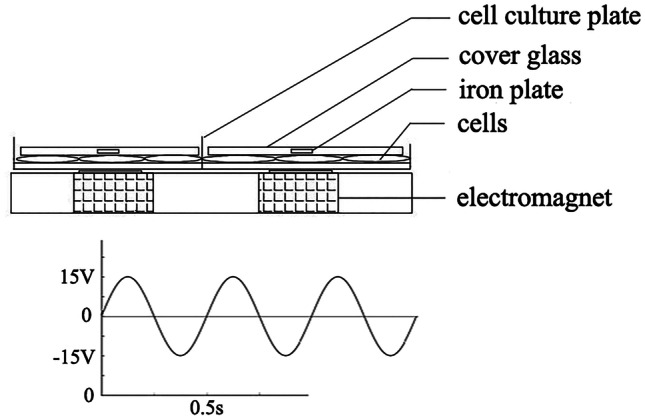
Schematic of the electronically controlled MS-generating device.

As in Fig. 1, briefly, a small round iron plate coated with thin cover glass is placed over the confluent cell layers in the 6-well plate. The cyclic MS was generated as magnetic force loaded on the iron plate by an electromagnet below the 6-well plate, which was modified from the device we had reported [37, 38]. The magnitude and frequency of the stress were controlled by an arbitrary waveform generator and a power amplifier which linked to the electromagnet. Cells of the control group were covered with a thin glass without the electromagnet energized below. The experiment of MS application was performed in a humidified atmosphere incubator containing 95% air and 5% CO_2_ at 37 °C. Although we did not measure the exact magnitude of the pressure on the chondrocytes below cover glass when MS loaded, we choose a sinusoidal waveform power of which the peak voltage and frequency were 15 V and 2 Hz, respectively, as eMS in this experiment, based on the results of our previous research (unpublished).

### Proteins Extraction and Western Blot

After treatment of MS, cells were washed with cold PBS and lysed in a lysis buffer (Beyotime, China). Lysates were mixed and incubated on ice for 15 min, and then, cell debris was spun down at a speed of 14,000 rpm for 15 min at 4 °C. Concentrations of protein samples in the supernatant were determined using the bicinchoninic acid (BCA) method with the protein assay kit (Beyotime, Shanghai, China). Equal quantities (20 μg) of proteins samples (dissolved in 5 × loading buffer) were separated using SDS-PAGE gels (5% stacking gel and a 10% running gel) and then electro-transferred to polyvinylidene fluoride (PVDF) membranes at 250 mA for 30 min. After blocking in 5% bovine serum albumin (BSA) in TBST at room temperature for 1 h, membranes were then incubated with primary antibodies (1:1,000 except for ERK (1:2,000) and β-actin (1:2,000)) overnight at 4 °C. After washing with TBST, membranes were then incubated with HRP-conjugated secondary antibodies (1:2,000) at room temperature for 1 h. Protein bands were visualized using ECL reagents. The intensity values of each phosphorylated kinase were quantified using densitometric analysis with ImageJ 1.36 and normalized to the intensity of corresponding total protein bands. Unless otherwise stated, β-actin was used as an internal control.

### RNA Extraction and qRT-PCR

To isolate total RNA from the articular cartilage, cartilage from the femoral condyles was shaved with a scalpel and subsequently frozen immediately in liquid nitrogen. Using the Bio-Gen PRO200 Homogenizer (PRO Scientific Inc., USA), the cartilage was homogenized in TRIzol (Invitrogen, USA) according to the manufacturer’s instructions. Then, the RNA was dissolved in 0.1% diethylpyrocarbonate water and quantified by spectrophotometry, and samples with values between 1.7 and 2.0 were used. A total of 1 µg RNA was used to synthesize complementary DNA (cDNA) by reverse transcription using a PrimeScript™ RT reagent kit (Takara, Japan) according to the manufacturer’s instructions. Subsequently, the samples were subjected to qPCR using SYBR Premix Ex Taq (TaKaRa, Japan) with specific primers (Table 1). The gene for β-actin acted as an endogenous reference for normalization of fluorescence thresholds (Ct) values of target genes.

**Table 1:** Primer sequence for qRT-PCR

Primer name	Sequence
*Collagen II* XM_006242308.4	F: 5′-CTGTCTGCAGAATGGGCAGAGG-3′ R: 5′-GCCAGGAGGTCCTTTAGGTCCT-3′
*Aggrecan* XM_039101034.1	F: 5′-AGGACAGGTTCGAGTGAACAGC-3′ R: 5′-GGTCAAAGTCCAGTGTGTAGCG-3′
*MMP-13* NM_133530.1	F: 5′-CTGGTCTGATGTGACACCTCTG-3′ R: 5′-CTTTGGAGCTGCTTGTCCAGGT-3′
*MMP-9* NM_031055.2	F: 5′-TTGTCCTGCACCACGGATGGCC-3′ R: 5′-ACCAGCGATAACCATCCGAGCG-3′
*Adamts-5* NM_198761.2	F: 5′-AGCGCAGCTGCGCTGTGATTGA-3′ R: 5′-TCTGTGATCGTGGCTGAAGTGC-3′
*Runx2* XM_039083955.1	F: 5′-GGCCTTCAAGGTTGTAGCCCTC-3′ R: 5′-TAGCTCTGTGGTAAGTGGCCAC-3′
*Integrin αV* XM_039106448.1	F: 5′-CGTCCTCCAGGATGTTCCTCCT-3′ R: 5′-GGCTCCAAACCACTGGTGGGAT-3′
*Integrin β3* XM_039085714.1	F: 5′-CGTCGGAGAGTCCAACATCTGT-3′ R: 5′-TGTCTCCTGAGCCCTTGCTGCT-3′
*β-actin* NM_031144.3	F: 5′-TCATGCCATCCTGCGTCTGGAC-3′ R: 5′-TGCCGATAGTGATGACCTGACC-3′

### Statistical Analysis

SPSS version 13 for Windows was used for all statistical analyses. All data were presented as mean ± standard deviation (SD). Differences among groups were compared using one-way ANOVA with Bonferroni post-hoc test. Differences between two groups were tested using paired-samples *t* test when appropriate. All experiments were repeated at least three times. *P* < 0.05 was considered statistically significant.

## RESULTS

### Upregulated Integrin αVβ3 In the Weight-Bearing Area of the OA Knee Joint

**Fig. 2:**
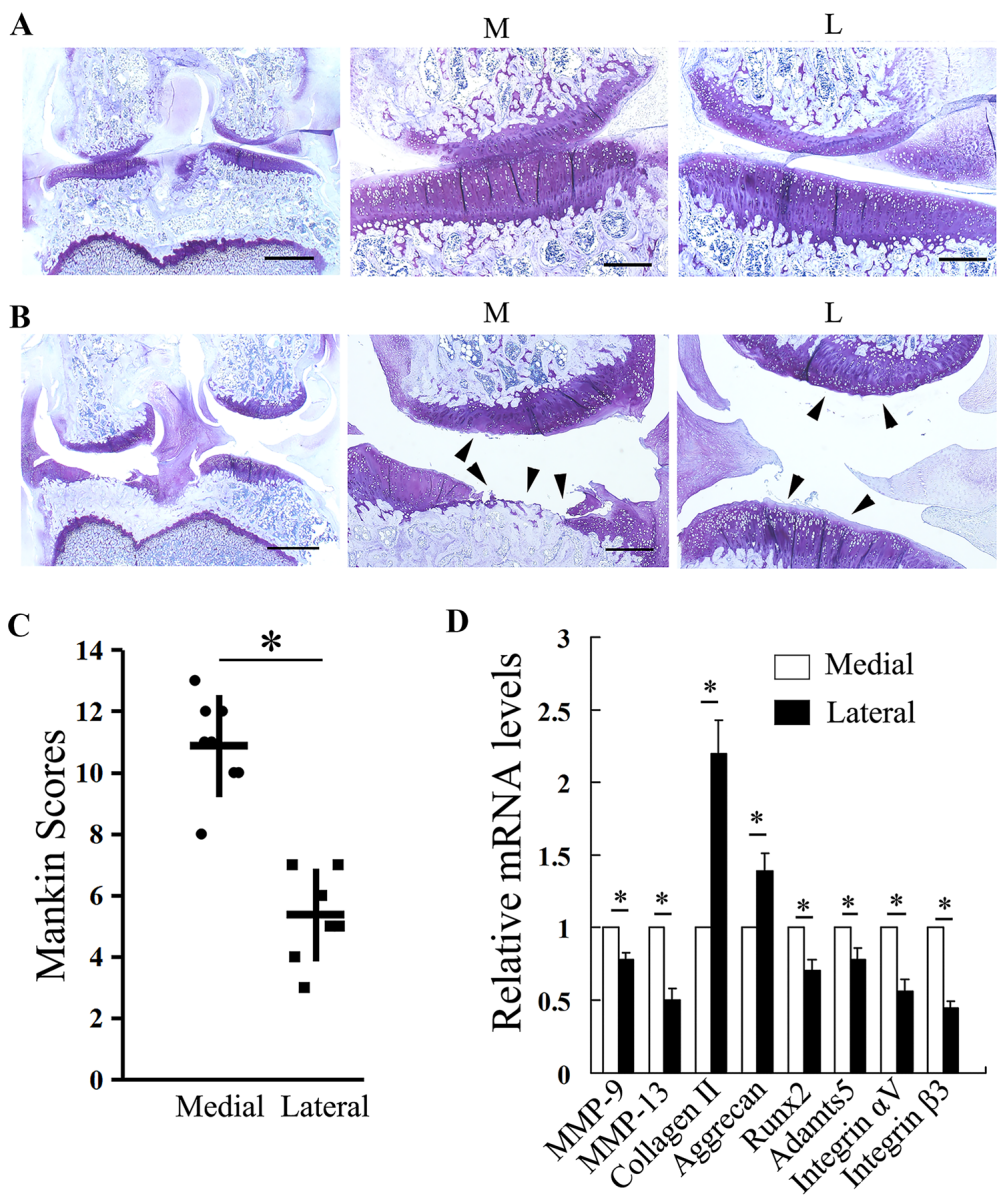
Different severity of OA in medial and lateral regions of rat knee joints and upregulated integrin αVβ3 in the weight-bearing area (**A** normal joint; **B** DMM joint). Histological observations of representative sections of the medial and lateral regions of rat joints stained with toluidine blue. Arrowheads indicate areas of cartilage degeneration. Scale bars in the low-magnification (left) images: 1 mm; scale bars in the high magnification (middle and right) images: 250 μm. **C** Quantification of cartilage degeneration in toluidine blue-stained sections of the medial and lateral regions of joints from rats 12 weeks after DMM surgery (*n* = 8). **D** qRT-PCR analysis of mRNA levels for MMP-9, MMP-13, collagen II, aggrecan, Runx2, Adamts 5, integrin αV, and integrin β3 in cartilage from the medial and lateral tibial plateau of rat knee joints 12 weeks after DMM surgery. Data are presented as mean ± SD of triplicates and are representative of 8 individual samples. *P < 0.05 compared with cartilage from the medial tibial plateau. M, medial; L, lateral.

Firstly, we tested the contribution of MS to OA progression in a rat DMM-induced OA model by comparing the differences between medial and lateral areas of the OA knee joint, where there were different MS conditions. Twelve weeks after DMM surgery, histological observation demonstrated normal cartilage of the left knees (Fig. 2A) and the medial area of the femoral condylar and tibial plateau exhibited severe cartilage loss and abrasion with loss of ECM, exposed subchondral bone, and irregular arrays and shape of chondrocytes compared with that of the lateral area in the right knees (Fig. 2B). The modified Mankin score was significantly higher in the medial area than that of the lateral area, which implied more severe OA in the weight-bearing area of the knee (Fig. 2C).

We also investigated the role of integrin αVβ3 in the pathogenesis of OA by examining OA-related inflammatory and degradative mediators in the rat OA model. Cartilage of the medial area of the OA knees exhibited elevated mRNA levels of integrin αV and β3, as revealed by qPCR analyses. Results of qPCR also demonstrated significantly increased expressions of MMP-9, MMP-13, ADAMTS-5, and Runx2 with decreased collagen II and aggrecan expressions at the medial area, compared with that of the lateral area (Fig. 2D). These results reflected that elevated integrin αV and β3 expressions in the weight-bearing area of the OA knee joint may play an important role in the MS-induced OA.

### Excessive MS-Induced Phosphorylation of FAK and ERK and Promoted the Production of Osteoarthritis-Related Inflammatory and Degradative Mediators in Rat Chondrocytes

**Fig. 3:**
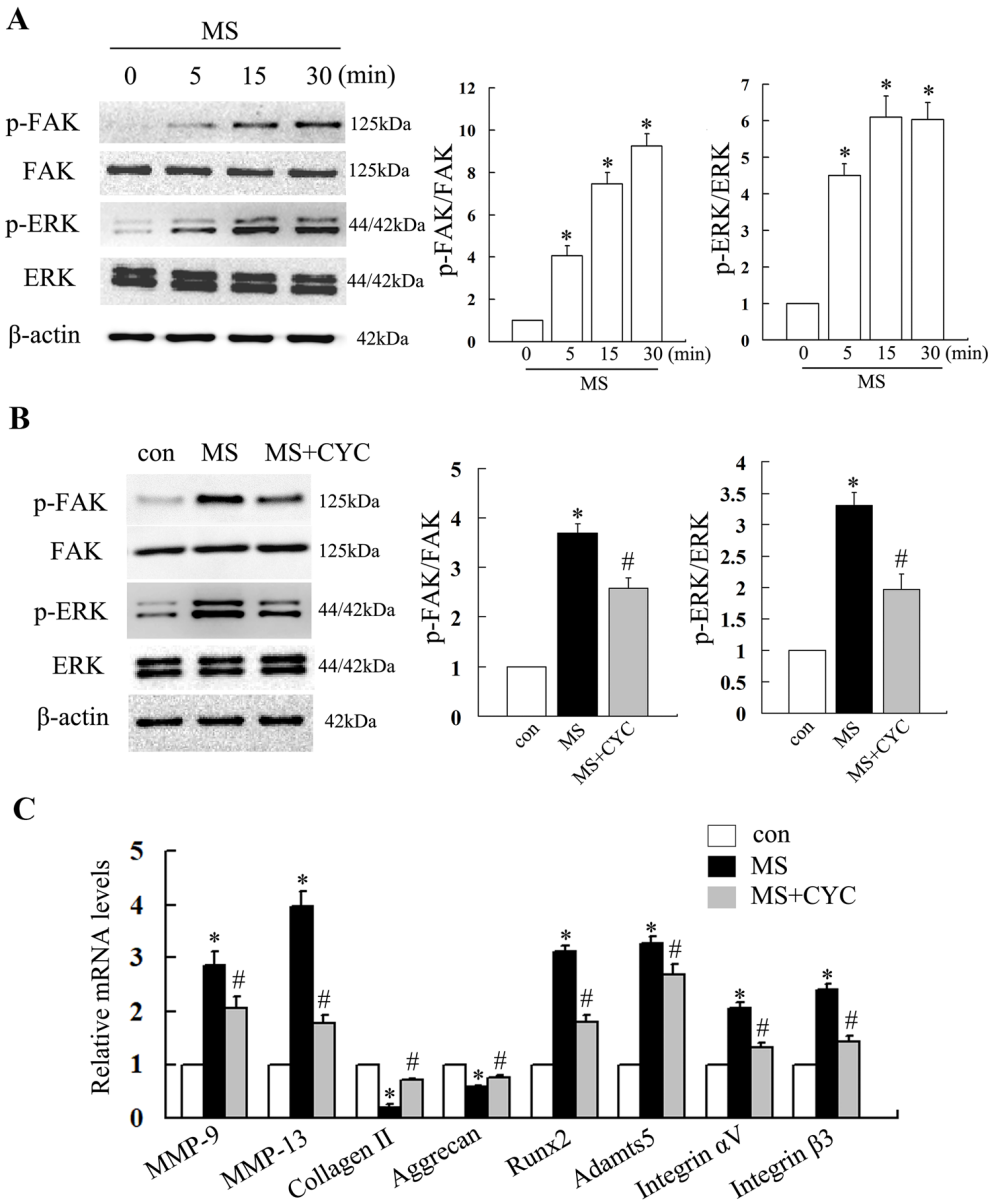
Crucial role of integrin αVβ3 in the development of rat osteoarthritis. **A** Western blot analysis of the phosphorylation of molecules downstream of integrin αVβ3 signaling in rat chondrocytes stimulated by eMS changes over time. **B** and **C** Integrin αVβ3-dependent phosphorylation of FAK and ERK and expression of inflammatory and degradative mediators induced by eMS. *P < 0.05 compared with control group. ^#^*P* < 0.05 compared with MS group.

To investigate the effect of eMS on rat chondrocytes, the downstream molecules of integrin αVβ3 signaling were evaluated by Western blot analysis, and the expression of osteoarthritis-related inflammatory, degradative, hypertrophic mediators as well as integrin αVβ3 was tested by qPCR analysis. The results of Western blot indicated that eMS led to rapid and transient phosphorylation of FAK and ERK over time. As the peak level of phosphorylated ERK occurred at 15 min and 30 min, while FAK phosphorylation increased approximately tenfold at 30 min compared with the control, we chose 30 min for the experiments in this report (Fig. 3A, B). qPCR analysis demonstrated that 24 h after MS stimulation, the expression of integrin αVβ3 was significantly increased. Meanwhile, the mRNA levels of MMP-13, MMP-9, ADAMTS-5, and Runx2 were increased, while the levels of collagen II and aggrecan were decreased (Fig. 3C). These outcomes implied that integrin αVβ3, FAK, and ERK were activated in chondrocytes in response to eMS stimulation, thereby inducing osteoarthritis-related inflammatory and degradative mediators production.

### Integrin αVβ3-Dependent Expression of Inflammatory and Degradative Mediators Induced by eMS

To investigate the role of integrin αVβ3 in eMS-induced chondrocytes inflammation, we pretreated the cells with the integrin αVβ3 inhibitor, CYC for 24 h to clarify whether the inhibitor had any effect on ERK and FAK phosphorylation induced by MS. The results showed that pretreatment with CYC significantly inhibited the expression of integrin αVβ3 in chondrocytes induced by eMS and attenuated eMS-induced increasing of ERK and FAK phosphorylation (Fig. 3B). The results of qPCR revealed that inhibition of integrin αVβ3 resulted in upregulation of collagen II, aggrecan expressions, and decreased expressions of MMP-13, MMP-9, ADAMTS-5, and Runx2 compared with cells treated with MS alone (Fig. 3C). These results indicated that elevated integrin αVβ3 expression in chondrocytes under eMS was a key factor in OA progression.

### Attenuation of Rat Osteoarthritis by Pharmacological Inhibition of Integrin αVβ3

**Fig. 4:**
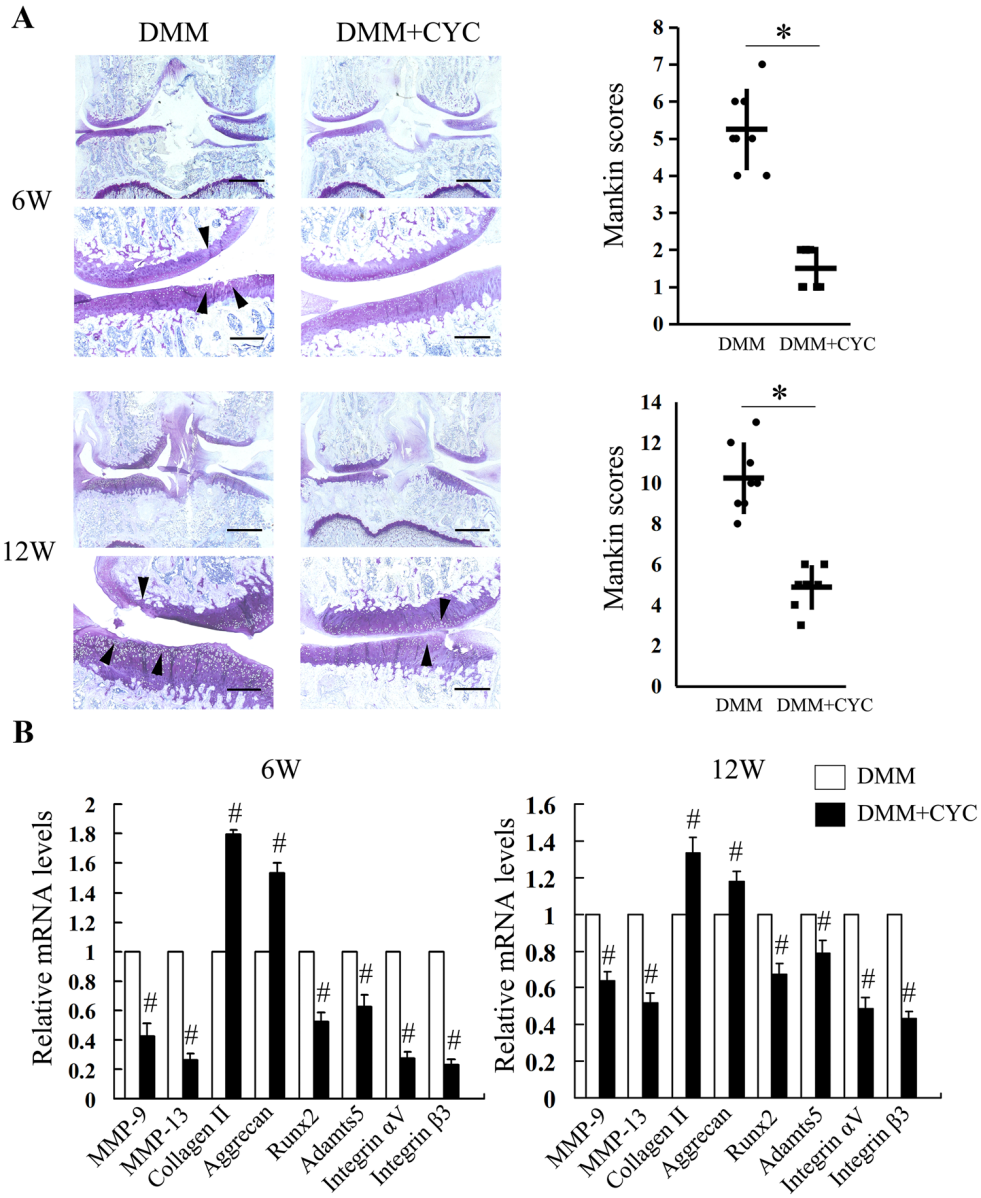
Attenuation of rat osteoarthritis by pharmacological inhibition of integrin αVβ3. **A** Histological observations of representative sections stained with Toluidine blue of the medial regions from rat joints 6 and 12 weeks after destabilization of the medial meniscus (DMM) and quantification of cartilage degeneration (*n* = 8). Arrowheads indicate areas of cartilage degeneration. Scale bars in the low magnification (left) images: 1 mm; scale bars in the high-magnification (middle and right) images: 250 μm. *P < 0.05 compared with DMM group. **B** qRT-PCR analysis of mRNA levels for MMP-9, MMP-13, collagen II, aggrecan, Runx2, Adamts 5, integrin αV, and integrin β3 in cartilage from the medial tibial plateau of rat knee joints 6 and 12 weeks after DMM surgery. Data are presented as mean ± SD of triplicates and are representative of 8 individual samples. ^#^*P* < 0.05 compared with DMM group. CYC, Cyclo (RGDyK).

To explore the inhibition of integrin αVβ3 in the treatment of OA induced by eMS, we treated the DMM rat model with knee joint injection of CYC. At 6 weeks after DMM surgery, the joints of the control group exhibited partial cartilage loss and abrasion without subchondral bone exposed in the medial area of the femoral condylar and tibial plateau, and the matrix surrounding the chondrocytes was partially lost. In the joints injected with CYC, the articular cartilage was smooth, without any evidence of injury or abrasion. The matrix surrounding the chondrocytes was arranged in columns that were smooth and evenly stained, just like the normal knee joint. Histologic analysis revealed that CYC-treated group exhibited a lower Mankin score than that of control (Fig. 4A).

The joints of the control group displayed severe cartilage abrasion, loss of extracellular matrix, irregular array and shape of chondrocytes, and even subchondral bone exposing 12 weeks after DMM surgery. The CYC-treated group exhibited reasonable cartilage regeneration compared with that of the control group, but still exhibited surface discontinuity, including shallow vertical fissures through the cartilage superficial zone at numerous points across the surface and delamination of the superficial zone. The Mankin score of the CYC-treated group was significantly lower than that of the control (Fig. 4A).

We also detected MMP-13, MMP-9, ADAMTS-5, Runx2, collagen II, aggrecan, and integrin αVβ3 expressions using qPCR analysis. At 6-week post-injection, higher levels of collagen II and aggrecan expressions were found in the CYC-treated group compared with that of the PBS-treated group. Furthermore, CYC injection led to significantly lower expressions of MMP-13, MMP-9, ADAMTS-5, and Runx2, compared with those of the PBS-treated group. At 12 weeks after CYC injection, qPCR analysis demonstrated downregulated MMP-13, MMP-9, ADAMTS-5, and Runx2 expressions and upregulated collagen II and aggrecan expressions in cartilage compared with the PBS group (Fig. 4B).

## DISCUSSION

Enormous studies have verified an important role of the meniscus in shock absorption and load distribution of knee joints [30, 39–41]. It has been extensively reported that medial meniscectomy could lead to stress concentration in the medial part of the knee joint which results in early degeneration of the joint [17, 30, 42, 43]. However, the molecular mechanisms underlying the inconsistent degree of medial and lateral cartilage OA in the knee after medial meniscus resection have not been fully researched. As a key receptor in the classical mechanotransduction pathway, integrins have been widely studied. In the present study, by examining the medial weight-bearing area of the knee after DMM surgery, we found a significant increase in the expression of integrin αVβ3 and OA-related inflammatory and degradative mediators in the cartilage of the medial weight-bearing area, which is consistent with the findings of Wang et al. [17]. They believed that the upregulation of integrin αVβ3 expression in chondrocytes was attributed to the inflammatory environment of the joint, and the upregulation of integrin αVβ3 further caused the aggravation of OA, forming a vicious circle. However, they did not investigate the different degrees of cartilage degeneration in the medial and lateral weight-bearing areas of the knee, nor did they take mechanical stress into consideration. Therefore, it seems difficult to make a perfect explanation to the phenomenon.

The effects of mechanical stress on chondrocyte inflammation and related signaling pathways have been extensively studied. It has been reported that moderate stress suppressed IL-1β-induced chondrocyte apoptosis by inhibiting the PI3K/Akt pathway [12]. However, Lohberger et al. found that in response to MS, human normal and OA chondrocytes showed different trends in Akt phosphorylation [33]. Our previous studies revealed that Akt phosphorylation could be regulated by different strengths of MS [37, 38]. The different results between the above reports may be attributed to the types and parameters of MS, cell source and culture methods, and even inflammation conditions [10]. The results of our present study demonstrated that eMS could induce the OA phenotype of chondrocytes. At the same time, the expression of integrin αVβ3 was significantly increased after eMS stimulation. The results of Naoto et al. demonstrated the upregulation of integrin (αVβ3 and αVβ5) expressions under MS in chondrocytes, which is consistent with our results [44].

FAK is a crucial signaling molecule and is initially stimulated by integrin activation, which can result in tyrosine (Tyr397) phosphorylation of FAK (depending on integrin-ECM interaction) and potentially activation of MAPKs under MS [45]. Then, the activated integrin-FAK-MAPKs axis will regulate OA-related factors such as MMPs, ADAMTs, IL- 6, and TNF-α [44]. In the present study, we also examined the signaling pathways above. Consistent with *in vivo* experiments, a significant increase of integrin αVβ3 expression as well as upregulated phosphorylation of FAK and ERK was found in chondrocytes after eMS stimulation, which is consistent with the report by Naoto et al. [44].

As a member of Runxs family, Runx2 plays an important role in bone mineralization by stimulating osteoblast differentiation [46]. Runx2 has also been reported to contribute to the pathogenesis of OA through upregulation of inflammatory factors and chondrocyte hypertrophy after the induction of joint instability [31]. These findings prompted us to investigate the effect of MS on Runx2 expression. Our findings showed that chondrocytes stimulated with eMS exhibited upregulated expressions of Runx2 and other OA-related inflammatory factors such as MMP-9, 13, and Adamts-5, indicating that eMS can exert a pro-inflammatory effect through integrin-FAK-ERK-Runx2 signaling.

Our data suggested that cartilage degeneration was closely associated with integrin αVβ3 expression and MS. However, different levels of cartilage degradation suggest that chondrocytes of medial and lateral parts with different ECM volume and quality can protect cells from the damage of inflammatory mediators [47]. Therefore, based on the results of animal studies, we cannot rule out the possibility of the influence of OA inflammation on integrin expression, just as reported by Wang et al. [17]. In order to figure out the role of integrin αVβ3 in the pathogenesis of MS-induced OA, we used CYC, a specific inhibitor of integrin αVβ3 [35, 48], to block transduction of MS in chondrocytes. The present study showed that inhibition of activated integrin αVβ3 led to a significant decrease in phosphorylation of FAK and ERK as well as cellular inflammation under eMS. Therefore, it can be preliminarily considered that eMS mediated chondrocyte inflammation through the upregulation of integrin αVβ3. Naoto et al. showed that inhibition of integrin αVβ3 and αVβ5 signaling with cilengitide significantly downregulated several inflammatory factors such as IL-1β, TNF-α, and MMPs and downregulated phosphorylation of MAPKs (ERK, JNK, and p38) in chondrocytes under eMS [44]. Cilengitide is a nonselective inhibitor of integrins that blocks the activation of both αVβ3 and αVβ5. In the present study, however, we modulated the integrin signaling with CYC, a specific inhibitor of αVβ3, to focus on the effects of this unique heterodimer on OA. Furthermore, *in vivo* studies showed that injection of CYC led to less cartilage damage and decreased expressions of OA-related inflammatory and degradative mediators in the medial weight-bearing area of the knee joint, indicating that inhibition of integrin αVβ3 delayed the development of OA, which we attributed this in part to the blocking of the integrin αVβ3-mediated MS transduction. Nevertheless, the role of αVβ3 in chondrocyte inflammation remains controversial, and some studies have suggested that αVβ3 plays a protective role in chondrocyte inflammation [22, 49, 50]. Lu et al. reported that IL-1β could induce chondrocyte inflammation, accompanied by a decrease in integrin αVβ3. Their study demonstrated that an increased expression of αVβ3 could inhibit inflammation in chondrocyte [50]. However, Attur et al. suggested that an inflammatory environment would lead to a high expression of integrin αVβ3 on the surface of chondrocyte, which could inhibit IL-1β transcription and attenuate inflammatory response [49]. Based on our findings, we believe that regulation of integrin αVβ3 on chondrocyte inflammation is an extraordinary complicated process, which needs further investigation.

Histological observation and qPCR analysis demonstrated that knee OA still progressed in the CYC-treated group at 12 weeks compared with 6 weeks postoperatively, suggesting that inhibition of integrin αVβ3 could not completely block the pathological process of OA, implying that other types of integrins in addition to αVβ3 and/or other signaling pathways may also participate in the pathogenesis of MS-induced OA. OA has always been considered a multifactorial disease with complex pathogenesis, and simply inhibiting one certain signaling pathway often fails to completely prevent the development of OA [34]. Our study provided preliminary evidence that integrin αVβ3, or at least partially, mediated eMS-induced OA via FAK-ERK-Runx2 signaling pathway.

One of the innovations of our study was to compare cartilage degradation in the medial and lateral weight-bearing areas of the same joint to illustrate the effect of different stress conditions on chondrocyte inflammation, and the results showed that integrin αVβ3 expression was positively correlated with the severity of OA. After excluding the effect of inflammatory mediators in the joint cavity on cartilage chondrocytes, we supposed that it is reasonable to attribute OA pathogenesis to MS. Additionally, we performed *in vitro* experiments using an electronically controlled MS-generating device of our own design, which has never been reported before. Due to the advantages of normal culture conditions and infinitely variable MS parameters, this device demonstrates the potential for future studies of cell biology in response to mechanical stimuli *in vitro*.

This study has multiple limitations. Firstly, we did not measure the exact value of the stress under the cover plate applied to the cells. The aim of this study was to elucidate the molecular mechanisms of chondrocyte inflammation under eMS instead of details about the exact values of stress. The effects of different types and strengths of MS on cells and the intracellular properties of mechanical transduction to MS still need further investigation. Secondly, we used CYC to inhibit integrin αVβ3 in animal studies, which ultimately delayed the development of OA. *In vitro* experiments confirmed that chondrocyte inflammation could be reduced after integrin αVβ3 inhibition under eMS. However, the animal study still has some shortcomings, as integrins have been reported to mediate the exacerbating effect of OA-related inflammatory mediators on chondrocyte inflammation [9, 16, 17, 51], attenuation of rat OA by pharmacological inhibition of integrin αVβ3 cannot be fully attributed to the blocking of high stress. Taken together, although a more complex situation exists in animal experiments, our data demonstrate that integrin αVβ3 plays an important role in eMS-induced OA. Future studies will be needed to clarify that the exact role of integrin αVβ3 signaling and to elucidate more specific molecular mechanisms in MS-induced OA will be needed to treat OA for the safe and effective treatment of OA.

In conclusion, we describe a signaling pathway linking eMS to cartilage degeneration. In the present study, we focused on the role of integrin αVβ3 in OA induced by eMS. We examined its expression in the medial and lateral parts of knee articular cartilage, its role in OA development *in vitro* and *in vivo*, and further downstream pathways linking eMS to cartilage inflammation. Upregulation of integrin αVβ3 induced by eMS in chondrocytes further activates downstream FAK-ERK signaling and alters chondrocyte metabolism to promote inflammation and degradation of ECM. Our findings suggest modulating the integrin αVβ3 signaling pathway as a potential disease-modifying therapy for OA.

## Data Availability

The data used and/or analyzed during this study are available from the corresponding author on reasonable request.

## Abbreviations

OA: Osteoarthritis
eMS: Excessive mechanical stress
ECM: Extracellular matrix
NF-κB: Nuclear factor kappa-light-chain-enhancer of activated B cells
DMM: Destabilization of the medial meniscus
FAK: Focal adhesion kinase
MAPKs: Mitogen-activated protein kinases
Runx2: Runt-related transcription factor 2
Adamts-5: A disintegrin and metalloproteinase with thrombospondin type-5 motif
CYC: Cyclo (RGDyK)
